# Plant-Based Synthesis of Gold Nanoparticles and Theranostic Applications: A Review

**DOI:** 10.3390/molecules27041391

**Published:** 2022-02-18

**Authors:** Uday M. Muddapur, Sultan Alshehri, Mohammed M. Ghoneim, Mater H. Mahnashi, Mohammed Abdulrahman Alshahrani, Aejaz Abdullatif Khan, S. M. Shakeel Iqubal, Amal Bahafi, Sunil S. More, Ibrahim Ahmed Shaikh, Basheerahmed Abdulaziz Mannasaheb, Noordin Othman, Muazzam Sheriff Maqbul, Mohammad Zaki Ahmad

**Affiliations:** 1 Department of Biotechnology, KLE Technological University, Hubbali 580031, India; 2 Department of Pharmaceutics, College of Pharmacy, King Saud University, Riyadh 11451, Saudi Arabia; salshehri1@ksu.edu.sa; 3 Department of Pharmacy Practice, College of Pharmacy, AlMaarefa University, Dariyah 13713, Saudi Arabia; mghoneim@mcst.edu.sa (M.M.G.); bmannasaheb@mcst.edu.sa (B.A.M.); 4 Department of Pharmaceutical Chemistry, College of Pharmacy, Najran University, Najran 66462, Saudi Arabia; matermaha@gmail.com; 5 Department of Clinical Laboratory Sciences, College of Applied Medical Sciences, Najran University, Najran 66462, Saudi Arabia; maalshahrani@nu.edu.sa; 6 Department of General Science, Ibn Sina National College for Medical Studies, Al Mahajar Street, P.O. Box 31906, Jeddah 21418, Saudi Arabia; aeju_kh@yahoo.com; 7 Department of Pharmaceutical Chemistry, Ibn Sina National College for Medical Studies, Al Mahajar Street, P.O. Box 31906, Jeddah 21418, Saudi Arabia; alibahfi@hotmail.com; 8 School of Basic and Applied Sciences, Dayananda Sagar University, Bangalore 560078, Karnataka, India; sunilsmorey@gmail.com; 9 Department of Pharmacology, College of Pharmacy, Najran University, Najran 66462, Saudi Arabia; i.ibrahimshaikh09@gmail.com; 10 Clinical and Hospital Pharmacy Department, College of Pharmacy, Taibah University, Al-Madinah Al-Munawwarah 41311, Saudi Arabia; nbinothman@taibahu.edu.sa; 11 Department of Clinical Pharmacy, School of Pharmacy, Management and Science University, University Drive, Off Persiaran Olahraga, Shah Alam 40100, Selangor, Malaysia; 12 Department of Microbiology and Immunology, Ibn Sina National College for Medical Studies, Jeddah 21418, Saudi Arabia; muashe@yahoo.co.in; 13 Department of Pharmaceutics, College of Pharmacy, Najran University, Najran 66462, Saudi Arabia; zaki.manipal@gmail.com

**Keywords:** gold, cytotoxicity, antioxidant, anticancer, antibacterial, antifungal

## Abstract

Bionanotechnology is a branch of science that has revolutionized modern science and technology. Nanomaterials, especially noble metals, have attracted researchers due to their size and application in different branches of sciences that benefit humanity. Metal nanoparticles can be synthesized using green methods, which are good for the environment, economically viable, and facilitate synthesis. Due to their size and form, gold nanoparticles have become significant. Plant materials are of particular interest in the synthesis and manufacture of theranostic gold nanoparticles (NPs), which have been generated using various materials. On the other hand, chemically produced nanoparticles have several drawbacks in terms of cost, toxicity, and effectiveness. A plant-mediated integration of metallic nanoparticles has been developed in the field of nanotechnology to overcome the drawbacks of traditional synthesis, such as physical and synthetic strategies. Nanomaterials′ tunable features make them sophisticated tools in the biomedical platform, especially for developing new diagnostics and therapeutics for malignancy, neurodegenerative, and other chronic disorders. Therefore, this review outlines the theranostic approach, the different plant materials utilized in theranostic applications, and future directions based on current breakthroughs in these fields.

## 1. Introduction

Gold nanoparticles (AuNPs), due to their unique qualities and various surface characteristics, have been widely exploited in bionanotechnology. The ease with which AuNPs can be functionalized makes them a flexible platform for nano biological assemblies containing oligonucleotides [1], antibodies [2], and proteins [3]. AuNPs bioconjugates have also emerged as attractive options for developing novel biomaterials for biomedical research. The versatility of AuNPs has made them useful in various biomedical applications. The binding of the sample to the AuNPs can change the rheological feature of AuNPs, such as surface plasmon resonance, conductivity, and redox behavior, resulting in notable signals [4,5,6,7] in diagnostics. With their enormous surface area, AuNPs can also be used as a platform for therapeutic agents. Nanotechnology has been in existence for thousands of years. Ancient people used to stain their drinking glasses with nanoparticles [8]. The divergence of nanotechnology within other fields of science and further innovations have made a significant impact on biotechnology, medicine, pharmaceutics, physics, chemistry, and optics, etc. There is evidence that metals are present in living systems in different forms, playing a significant role in various biochemical processes, growth metabolism, and healing [9]. Blood contains the Hema protein, Zn, Mn, Cu, and other vital trace metals in the biological system. 

This review aims to summarize the data on gold nanoparticles synthesized by extracts of medicinal plants, their parts, and their usefulness in biological and theranostic properties. 

### 1.1. Nanoparticle Synthesis

Nanoparticle synthesis from metals has gained enormous interest among researchers because of nanoparticles’ diverse application in many fields such as cancer therapy, drug delivery, food safety, fabrics, chemistry, water treatment, and photocatalysis, as well as because of their antioxidant, antibacterial, and cytotoxic properties [10,11,12,13,14,15,16,17,18,19,20]. The uses of nanoparticles in various fields are possible because of several factors, including the nanoparticles′ shape, size, distribution, and surface plasmon [21,22,23]. 

Nanoparticles have been used for thousands of years without knowledge concerning the exact phenomenon and synthesis [8]. Drinking glasses in ancient times were coated with Au nanoparticles and were synthesized following three primary methods: physical, chemical, and biological methods.

### 1.2. Physical Method

The advantages of the physical synthesis method are the absence of a solvent, which is hazardous to the environment, and the uniformity of the nanoparticles produced by the physical methods. The tube furnace method of synthesis occupies an ample space, and an enormous quantity of heat is required to raise the temperature of the furnace. Several minutes are necessary to preheat the furnace [24]. A small ceramic heater with a local heating chamber could be used to synthesize Ag nanoparticles [25]. As a result, the formed nanoparticles were reported to have a mean geometric diameter that was spherical without agglomeration [26]. The advantage of the laser ablation method, in comparison to other techniques, is that it is free from chemical reagents. The purity of the nanoparticles was assured in this method [27]. The nanoparticles produced by the discharge method used to fabricate Ag nanoparticles [28,29] had 99.99% purity. The purity and size distribution were uniform when compared to other forms of synthesis.

### 1.3. Chemical Synthesis of Gold Nanoparticles

In recent years, a solution-based strategy for controlling the size, shape, and surface functionality has been created [30,31,32]. In 1951, a new method for synthesizing AuNPs was devised by boiling hydrogen tetrachloroaurate (HAuCl_4_) with citric acid [33]. Citrate has a lowering and stabilizing effect [34]. To adjust particle size, Frens developed the process by modifying the gold-to-citrate ratio [33]. This approach has been commonly used to make dilute solutions of relatively stable spherical AuNPs with diameters of 10 to 20 nm; however, bigger AuNPs (e.g., 100 nm) have also been made. These citrate-stabilized AuNPs may undergo irreversible aggregation during the functionalization process with thiolate ligands. Several solutions have been devised to tackle this difficulty, including using a biosurfactant, Tween 20. Similarly, a two-step method for functionalizing gold nanoparticles was made by reducing tetrachloroauric acid in water with trisodium citrate. The physisorbed chloride and citrate on gold nanoparticles are first displaced by thioctic acid, which is then replaced by thiols with the desired functionality in the second step [35,36]. The demand for high dilution, on the other hand, makes large-scale manufacture difficult.

AuNPs synthesis [37] was conducted in 1994 to produce organic soluble alkanethiol-stabilized AuNPs by adopting a biphasic reduction, with the use of tetraoctylammonium bromide as a phase transfer reagent and sodium borohydride (NaBH_4_) as a reducing agent [37]. By changing response variables such as the gold-to-thiol ratio, the reduction rate, and the reaction temperature, this technique yields low-dispersity AuNPs ranging from 1.5 to 5 nm [38]. The synergic impact of thiol-gold generated strong connections and Van der Waals attractions between the adjacent ligands, giving these alkanethiol-protected AuNPs better stability than most other AuNPs [39]. 

## 2. Biological Method of Synthesis

**Figure 1:**
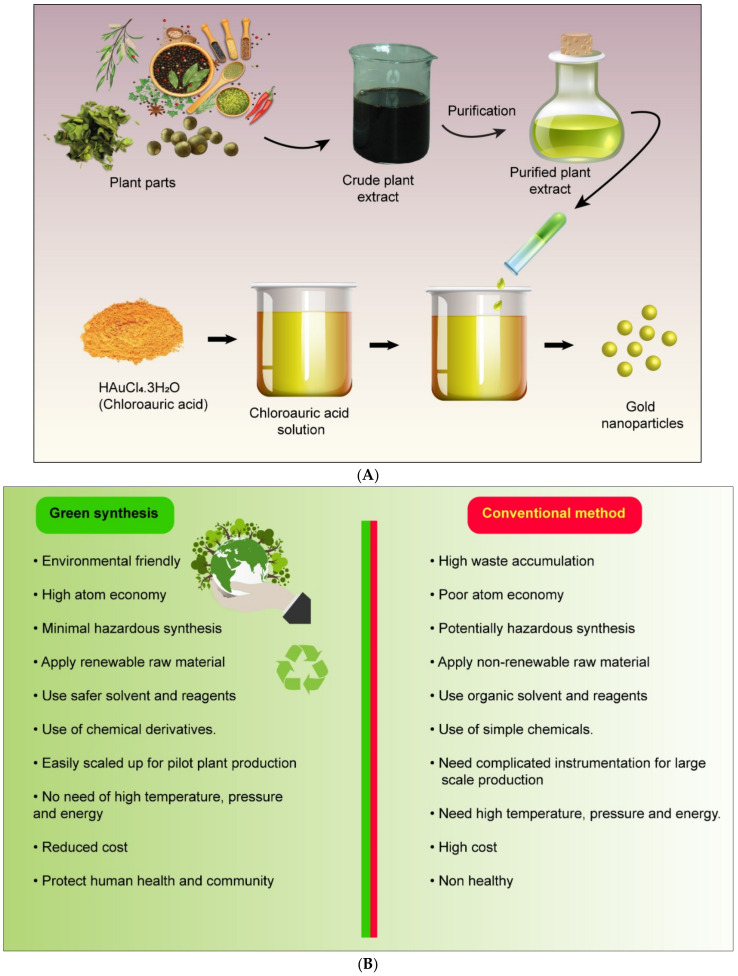
(**A**) Proposed synthetic mechanism for plant-mediated synthesis of gold nanoparticles. (**B**) The advantages of green synthesis over conventional methods.

Although the chemical synthesis of metallic nanoparticles is a standard procedure, the cost and hazardous effects of reducing reagents and stabilizing agents restrict their use. Furthermore, in biomedical applications, these nanoparticles could be toxic [40,41]. As a result, ecologically friendly and cost-effective nanoparticle synthesis techniques that do not rely on harmful chemicals are needed. In recent years, biological nanoparticle production has gained popularity as a green and environmentally friendly process [42]. Plants or plant extracts and microorganisms and enzymes were employed to synthesize nanoparticles using a natural method [43,44]. The proposed synthetic mechanism for plant-mediated synthesis of gold nanoparticles is depicted in Figure 1A.

Plants are increasingly being used to synthesize nanoparticles because of their widespread availability, low cost, environmental friendliness, and non-toxic nature. Plants such as *Azadirachta indica* have recently been used to study the production of AuNPs. *Medicago sativa*, *Aloe vera*, *Cinnamomum camphora*, *Pelargonium graveolens*, *Coriandrum sativum*, *Coriandrum sativum*, *Lemongrass*, *Terminalia catappa*, and *Terminalia catappa* have all been reported [41,45,46,47,48,49,50,51].

Many scientists are experimenting with the production of AuNPs from plant extracts as biomedicines against drug-resistant bacteria. Arunachalam et al., 2013, proposed using *Memecylon umbellatum* nanoparticles as chemical sensors [52]. Kalishwaralal et al., 2010, showed how a bacterium, *Brevibacterium casei*, can synthesize and stabilize spherical-shaped Au and Ag nanoparticles in an unprecedented green process. The biological activities of the produced particles were confirmed based on their durable anti-coagulant actions. Similarly, *Citrus limon*, *Citrus reticulata*, and *Citrus sinensis*, all citrus fruits, as well as *Piper pedicellatum*, have been synthesized as polymorphic gold nanoparticles with promising biological uses. These chemical constituents can operate as a reducing, stabilizing, and capping agent [52,53,54,55,56,57]. *Chebula Terminalia*, *Memecylon edule*, and *Nyctanthes arbor-tristis* flower extract have potential medicinal and industrial applications. *Murraya koenigii* and *Musa paradisiaca* show antibacterial activity; *Mangifera indica*, *Cochlospermum gossypium,* and *Cinnamomum zeylanicum* photoluminescent particles are used for the production of noble metal nanoparticles, which enable much faster synthesis and colloidal stability comparable to those of chemical reduction [58,59,60,61,62,63,64,65,66,67].

### 2.1. Green Synthesis of Gold Nanoparticles

Many metal nanoparticles synthesized via the green process possess several advantages, as shown in Figure 1B. Their unique physicochemical properties, high surface-to-volume ratio, low cost of synthesis, and surface functionalization were reported by Ankit Kumar Singh. Additionally, this review found that several studies have reported in detail a variety of plants and plant parts used in metal nanoparticle generation: the bark of *Mimusops elengi* was used to synthesize Au nanoparticles; bimetallic nanoparticles were synthesized using *Azadictira Indica* leaf extract; Au nanoparticles were synthesized from natural rubber; and *Aelovera* plant extract and lemongrass extract have applications on infrared-absorbing coating [68,69,70,71,72,73]. The antioxidant, anti-inflammatory, antidiabetic, and antibacterial activities of *Holopetelea integrifolia* leaf extract were studied, and synthesized Au nanoparticles from *Halymenia dilatata* were studied regarding their antioxidant, anticancer, and antibacterial activities; synthesized conjugated Au nanoparticles from *Nerium oleander* were studied regarding their anticancer activity against MCR-7 cell lines [74,75,76]. The anticancer activity of Au nanoparticles synthesized using *Lonicera japonica* was also studied. Ag and Au nanoparticles synthesized from *Pleuropterus multiflorus* roots were investigated regarding their anticancer activity against the A549 lung cancer cell line [77,78]. Au nanoparticles synthesized using the *Mucuna pruriens* plant extract were studied regarding an antiparkinsonian drug, and it was reported that poly-shaped Au nanoparticles were synthesized using *Saraca indica* bark extract and were studied regarding catalytic reduction. The anticancer activity of Ag and Au nanoparticles synthesized using Dendropanax morbifera leaf extract was studied, as well as the antimicrobial characteristics of Au and Ag nanoparticles using *Trianthema decandra* extract. The antioxidant and anticancer properties of Au nanoparticles synthesized using *Antigonon leptopus* leaf extract were studied, and the anticancer activities of noble metal nanoparticles using *Psidium guajava* leaf extract and *Syzygium aromaticum* bud extract were studied. The antibacterial properties of Au nanoparticles synthesized from *Nepenthes khasiana* leaf extract were investigated, as well as Au nanoparticles synthesized from *Schisandra Chinensis* fruit extract. Ag and Au nanoparticles synthesized using *Dalbergia sissoo* leaf extract were studied, and Ag nanoparticles synthesized from *Cassia italica* leaf extract were also studied. The kinetics of the Au nanoparticles synthesized using *Camellia chinesis* leaves and leaf buds were studied, as well as the apoptotic effects of Au nanoparticles synthesized using *Curcuma wenyujin* [79,80,81,82,83,84,85,86,87].

### 2.2. Medicinal Plants

Nature’s contribution to the health of human beings is unimaginable. A wide variety of plants are used in curing diseases and for a healthy lifestyle. India has a rich source of medicinal plants used for various purposes. More than 17,000 species are used as medicinal plants in India. The constituents/drugs present in medicinal plants are called phytochemicals. These phytochemicals act on the biochemical processes in animals, human beings, and microbes. The properties of phytochemicals are used due to their antioxidant, antimicrobial, and anti-inflammatory properties [88,89,90]. 

The World Health Organization (WHO) indicated that traditional remedies are used by 80% of the world’s population. For a long time, plants have been used as medicine in India’s alternative medical systems, such as Unani, Ayurveda, Siddha, Yoga, and homeopathy. Plant-derived medicines are alternatives to synthetic drugs, gaining importance in modern medicine. In the developing world, primary health care services have benefited from medicinal plants. In the Ayurvedic medical system, many plants and plant-based materials are employed to treat ailments. A treatise on Ayurvedic medicine called “Charaka Samhitha” mentions over 700 herbs [91,92,93,94,95,96,97]. Several therapeutic plants are mentioned in the Vedas, such as the Rig Veda and the Atharva Veda.

**Figure 2:**
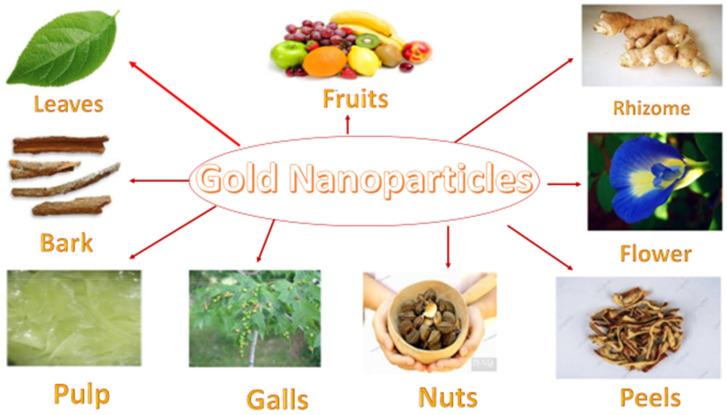
Plant parts extract can be used for the biosynthesis of gold nanoparticles.

AuNPs are well-known nanomaterials with a wide range of biomedical applications. AuNPs can be synthesized using a variety of microbes and plants, mainly through the use of fruit extracts. Fruit extracts are used because they naturally concentrate chemicals with medicinal effects. Studies have shown that UV–visible spectroscopy, transmission or scanning electron microscopy, dynamic light scattering, and Fourier transformation infrared spectroscopy techniques are the methods most often used to characterize AuNPs and capping biomaterial. Figure 2 shows some of the important outcomes in gold nanoparticles obtained from plant components.

## 3. Characterization

### 3.1. UV–Visible Spectroscopic Analysis 

**Figure 3:**
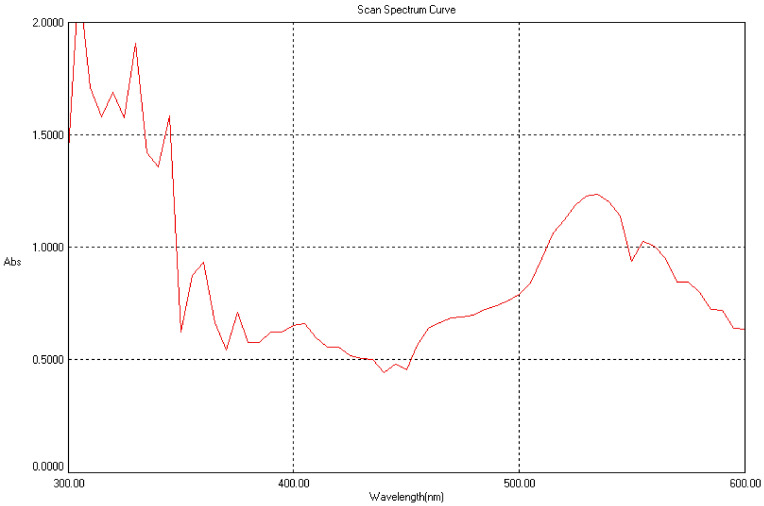
UV-spectral analysis for gold nanoparticles.

In an aqueous solution, gold nanoparticles synthesized from various plant parts were measured using a UV photometer and a Lab India UV3000 spectrophotometer, which read at 450 nm and 650 nm for the Au nanoparticles. Readings were taken every 30 min for 6 h. The absorbance and transmittance of the Au nanoparticles were measured at 450 nm to 650 nm using 3 mL of each sample in a cuvette, and they were subjected to spectral analysis. At 520–560 nm, a single, narrow absorbance band was found, which is typical of the production of tiny gold nanoparticles [98,99,100,101,102,103], and this was validated by the TEM results as shown in Figure 3.

### 3.2. Fourier Transform Infrared Spectroscopic Analysis (FTIR)

**Figure 4:**
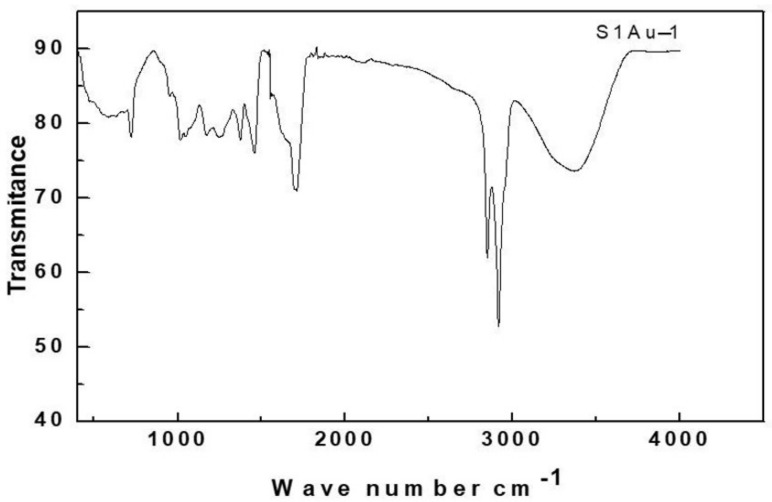
FTIR for gold nanoparticles.

A total of 5 gm of each synthesized freeze-dried Au nanoparticle from different plants parts was taken and pressed with 0.2 gm of KBr pellets to measure the infrared radiation spectrum (IR) examined under an FTIR spectrophotometer (JASCO) over wavelengths in the range of 4000 cm^−1^–400 cm^−1^. The FTIR spectrum of the green synthesized AuNPs is shown in Figure 4. The strong bands at 3389 cm^−1^ (O-H stretching alcohol), 2919 cm^−1^ (C-H stretching alkane) and 2844 cm^−1^ (C-H stretching aldehyde) were due to the reduction of Au^3+^ to Au0. A band at 1458 cm^−1^ corresponds to an NH bend, and the very broad band of NH^+3^ stretch was observed in the 3000–3500 cm^−1^ range. The peaks at 1700 cm^−1^ (C-C stretching alkane), 1374 cm^−1^ (O-H bending phenol), and 1162 cm^−1^ (CO-O- CO stretching anhydride) confirm the capping biomaterials of phytochemicals from plant extracts such as polyphenols, flavonoids, and terpenoid compounds. Similar reports of FTIR peaks for phenols and flavonoids from gold nanoparticles biosynthesized from *Cissus quadrangular* extract confirm the capping biomaterial of the synthesized nanoparticles. The bands at 1261 cm^−1^ and 1034 cm^−1^ are typically assigned to the vibration of ribose (C-C sugar), which correspond to an epoxy bond, semi-acetal, and primary alcohol, respectively. Further, the bands at 2920 and 1374 cm^−1^ correspond to methylene stretching and methyl deformation vibrations, respectively.

For example, the gold nanoparticles produced using plant extracts had bands at 617 cm^−1^, 1125 cm^−1^, 1376 cm^−1^, 1658 cm^−1^, and 3278 cm^−1^ in their FTIR pattern [104,105,106,107,108,109]. The aromatic hydroxyl and benzene rings were assigned bands at 3402 cm^−1^, 1606 cm^−1^, and 1518 cm^−1^, indicating that the extract contains phenols. The bands at 2931 cm^−1^ and 1402 cm^−1^ correspond to methylene stretching and methyl deformation vibrations, respectively, whereas the sugar content is shown by bands at 1260 cm^−1^, 1113 cm^−1^, and 1076 cm^−1^, which correspond to an epoxy bond, semi-acetal, and primary alcohol, respectively.

### 3.3. Transmission Electron Microscope (TEM) 

**Figure 5:**
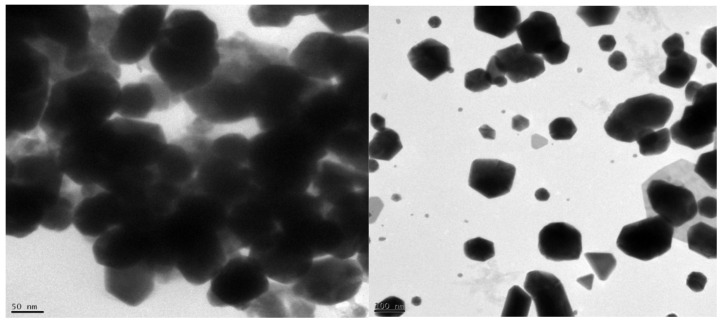
TEM analysis for gold nanoparticles.

The synthesized Au nanoparticles were loaded separately into the FEI. A Tecnai G2 F20 STFE-TEM microscope was used. The sample was dried by pressing with blotting paper to remove excess water and loaded onto the carbon-coated copper grid. The TEM was operated at 200 Kv, with a resolution of 0.24 nm, and Cs of 1.2 nm; the shape and size were determined as shown in Figure 5. The high-resolution TEM images show agglomerated polycrystalline particles, and the SAED confirmed the face-centered cubic (FCC) structure incorporation of the poly-dispersed XRD pattern. The EDX analysis proved the presence of only Au metal, and no other elements were present. 

SEM, TEM, and AFM are the most commonly utilized microscopic techniques for morphological analyses of nanoparticles. The application of these microscopic methods in nanoparticle morphology research has already been mentioned. TEM has a higher magnification and resolution than the SEM. The electron diffraction pattern for a specified region (SAED) is also utilized in TEM to distinguish crystalline structures from amorphous structures [105,110]. The shape of the gold nanoparticles is studied using AFM [109,110,111].

### 3.4. X-ray Diffraction (XRD)

**Figure 6:**
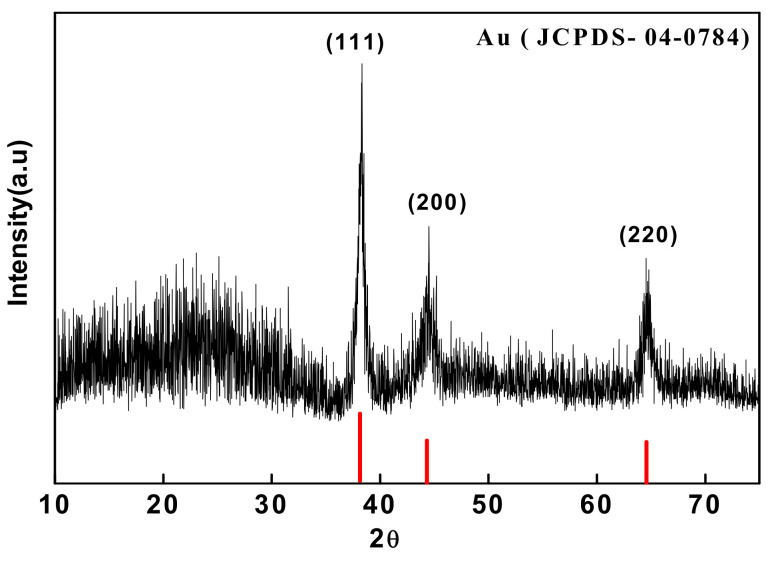
XRD analysis for gold nanoparticles.

The Analytical Expert MRD, the model instrument, is generally utilized to investigate the characteristics of synthetic Au nanoparticles for samples. The fine powder of nanoparticles is loaded onto the XRD sample holder separately, and readings are recorded. The size of the Au nanoparticles is calculated using Debye-Scherer’s equation: D = 0.9λ/βcos θ, where D is the average crystallite size. X is the XRD wavelength (1.54 nm); Β is the (FWHM), and θ is the Bragg angle. The plant-mediated synthesized XRD characterized the Au nanoparticles. The diffraction peak 2θ values assigned at 38.2º, 44.4º, and 64.6º were denoted as the (111), (200), and (220) planes, respectively. The planes agree well with the JCPDS card: 04-0784 data. The XRD pattern determined the intensity of the peak, the peak position, the width, and the full width at half-maximum (FWHM) as shown in Figure 6. The XRD data revealed that the nanoparticles are crystalline and face-cantered cubic (fcc). The particle mean size was determined using Debye-Scherer’s formula to determine the average size of the Au particles. The high-energy X-rays can penetrate the materials deeply and reveal important details about the bulk structure. The Debye-Scherrer equation computes the crystallite sizes using the XRD technique. The usage of XRD patterns/peaks during gold nanoparticle production has been reported [98,104,110,111,112,113,114]. 

## 4. Theranostic Applications

**Figure 7:**
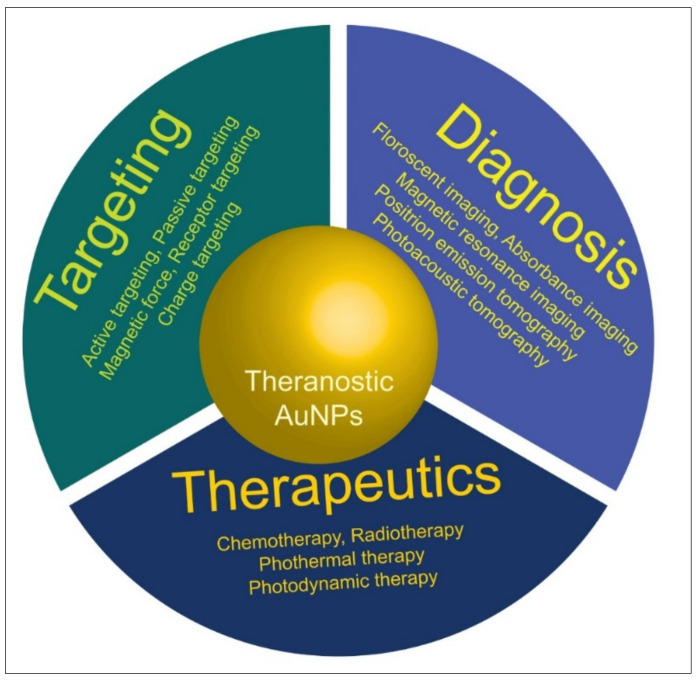
Theranostic applications of AuNPs in medical sciences and applied fields.

Nanoparticles are associated with a small stature and shape. The surface-to-volume ratio of nanoparticles is very high, which leads to enhanced electrical, optical, magnetic, antifungal, antioxidant, antibacterial, anti-inflammatory, and anticancer properties [115,116]. The surface-to-volume ratio offers many perspectives for the food sector. Nanoparticles’ are critical and significant in applications in biomedicine, especially in treating cancer, the diagnosis of HIV, and the proliferation of cancer cells. In 1918, scientists made drastic progress in finding the function of various metal nanoparticles in biological systems [117,118,119]. Metal nanoparticles, mainly gold, are used in medicine for diagnosis, targeting, and therapeutics (Figure 7). 

### 4.1. Principle of MTT Assay

**Figure 8:**
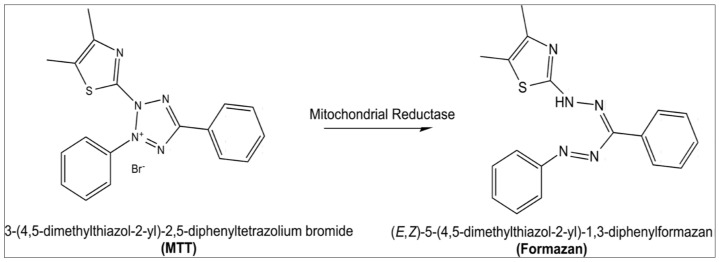
Principle of MTT assay.

Tetrazolium salt reduction is now universally acknowledged as a reliable method of examining cell growth. MTT (3-(4, 5-dimethylthiazolyl-2)-2, 5-diphenyltetrazolium bromide) is a yellow tetrazolium reduced by metabolically active cells, in part via dehydrogenase enzymes, to generate reducing equivalents such as NADH and NADPH. The intracellular purple formazan that results can be solubilized and measured using spectrophotometric methods (Figure 8). The assay evaluates the cell proliferation rate and, conversely, cell viability reduction induced by metabolic processes such as apoptosis or necrosis [120].

### 4.2. Biological and Theranostic Applications

As shown in Table 1, many researchers have demonstrated that AuNPs can successfully attack cancer cells. AuNPs derived from Gymnema Sylvestre, often known as cowplant, were cytotoxic to Hep2 cells. After treatment with AuNPs, Hep2 cells showed morphological alterations. Increases in reactive oxygen species levels and alterations in the nucleus were discovered, implying that apoptosis was responsible for the demise of Hep2 cells [121]. Another cervical cancer cell type, the HeLa cell line, also reacted to AuNPs. Rounding, shrinkage, and granulation were identified as morphological alterations. The AuNPs’ activity was attributable to the NPs’ ability to penetrate the cell membrane efficiently. AuNPs have caused responses from other tumor cells, including Ehrlich’s ascites carcinoma, breast cancer cells, and MCF-7 cells. Green tea polyphenols were used in the production of AuNPs. AuNPs synthesized from green tea and AuNPs synthesized from epigallocatechin-3-gallate were compared. Both AuNPs were able to trigger apoptosis in tumor cells while preventing tumor cell damage in normal hepatocytes. Green-generated AuNPs, on the other hand, demonstrated improved tumoricidal and hepatoprotective effects. When AuNPs generated by *Actinidia deliciosa* were examined on HCT-116 cells using an MTT assay, they showed 71 percent activity at their highest concentration (350 g/mL). The cytotoxic effect of the AuNPs was shown to be concentration dependent [122].

It is a known fact that using plants to make gold nanoparticles can result in nanoparticles with distinct biological properties. In a recent study by Mobaraki et al., 2021, using *Achillea biebersteinii* flower extract, spherical-shaped (8 nm) gold nanoparticles with anticancer properties against human testicular embryonic carcinoma stem cells were synthesized. The nanoparticles demonstrated dose-dependent cell viability against cancer cells by inducing apoptosis, with half inhibitory concentration (IC50) values of 10 g/mL [192]. In another study, Mousavi-Kouhi et al., 2022, synthesized gold nanoparticles from *Verbascum speciosum*; the green synthesized AuNPs were about 118 ± 72 nm in size and very effective against the hepatocellular carcinoma cell line (HepG2) and pathogenic bacteria [193]. 

Researchers are increasingly interested in the use of naturally occurring materials in biomedicine, and gum tragacanth (GT) has recently shown great promise as a therapeutic substance in tissue engineering and regenerative medicine. GT is a polysaccharide that can be extracted easily from the stems and branches of various Astragalus species. This anionic polymer is biodegradable, non-allergenic, non-toxic, and non-carcinogenic. GT′s resistance to microbial, heat, and acid degradation has made it a popular material in industrial (e.g., food packaging) and biomedical applications (e.g., drug delivery). GT has been shown to be a useful reagent in the formation and stabilization of metal nanoparticles over time [194,195].

## 5. Future Prospective

When we use green synthesis to make AuNPs, the process is simple. The reaction occurs in a controlled atmosphere with minimal temperature and pressure changes. Their reduction property determines the answer. A plant-based bioactive molecule that functions as a reducing agent usually produces the quickest reaction. Because of the benefits of employing green synthesis, we need to determine which molecules are feasible and to scale-up the commercialization of gold nanoparticles, as well as conduct the research needed for theranostic applications and disease markers. In addition, research should focus on in vivo investigations so that AuNPs can be used further as a medication or carrier for biomedical applications.

## 6. Conclusions

Diverse medicinal plants and their parts are employed to synthesize AuNPs, which have the unique virtue of having anticancer, antibacterial, and antifungal properties with theranostic applications. Nanotheranostics is a rapidly growing research field with enormous potential for improving disease diagnosis and treatment. Green nanoparticle synthesis, with its low capital requirements and operating costs, reduced pollution, and improved biocompatibility and stability, is a new and emerging field with advantages over chemical and physical nanoparticle synthesis methods. The number of biomedical applications in this sector is growing every day, with bioimaging, drug delivery, biosensors, and gene delivery among them. We hope that by focusing the readers’ attention on naturally synthesized nanoparticles and their applications, this review will help form a new perspective.

## Figures and Tables

**Table 1:** Biological and theranostic applications of gold nanoparticles.

S. No	Name of the Plant	Activity	Cell Line Used	Shape	**Size (nm)**	**Ref.**
1	Nanoparticles with antibacterial activity					
1.1	*Areca catechu*	Antibacterial	-	Spherical	13	[123]
1.2	*Acorus calamus*	Antibacterial	-	Spherical	100	[124]
1.3	*Ananas comosus*	Antibacterial	-	Spherical	16	[125]
1.4	*Benincasa hispida*	Antibacterial	-	Spherical	23	[126]
1.5	*Brazilian red propolis*	Antibacterial	-	Rods, triangular, pentagonal, hexagonal	8–15	[127]
1.6	*Clitoria ternatea (Asian* *pigeonwings)*	Antibacterial	-	Spherical, triangular, hexagonal	10	[128]
1.7	*Citrus maxima*	Antibacterial	-	Spherical	27–30	[104]
1.8	*Coreopsis lanceolate*	Detections of aflatoxins	-	Sphere	23–30	[129]
1.9	*Caesalpinia pulcherrima*	Antibacterial	-	Spherical	10–50	[130]
1.10	*Carthamus tinctorius L*	Antibacterial	-	Triangular, spherical	40–200	[131]
1.11	*Catharanthus roseus*	Antibacterial	-	Spherical, triangular	3–9	[132]
1.12	*Carica papaya*	Antibacterial	-	Spherical, triangular	2–20	[133]
1.13	*Coleus forskohlii*	Bactericidal activity	-	Triangular	25–40	[134]
1.14	*Ceiba pentandra (L)*	Antibacterial	-	Spherical	20–48	[135]
1.15	*Diospyros ferrea*	Antibacterial	-	Diverse	70–90	[136]
1.16	*Dioscorea batatas*	Antibacterial	-	Diverse	19–56	[137]
1.17	*Dimocarpus longan*	Antibacterial	-	Diverse	25	[138]
1.18	*Dracocephalum kotschyi*	Antibacterial	-	Spherical	11	[139]
1.19	*Euphorbia hirta*	Antibacterial	-	Spherical	6–7	[140]
1.20	*Gloriosa superba*	Antibacterial	-	Spherical	25	[141]
1.21	*Galaxaura elongate*	Antibacterial	-	Rod, triangular, hexagonal	3–77	[142]
1.22	*Bay cedar*	Antibacterial	-	Spherical	20–25	[143]
1.23	*Hibiscus cannabinus*	Antibacterial	-	Spherical	13	[144]
1.24	*Hoveniadulcis*	Antibacterial	-	Spherical	20	[145]
1.25	*Helianthus annuus*	Antibacterial	-	Polydispersed	35	[146]
1.26	*Hevea brasiliensis*	Cytotoxicity and genotoxicity	CHO-K1 cells	Spherical, triangular	50	[147]
1.27	*Justica wynaadensis*	Antibacterial	-	Spherical	30–50	[148]
1.28	*Jasminum auriculatum*	Antibacterial	-	Spherical	8–37	[149]
1.29	*Lobila nicotianifolia*	Antibacterial	-	Spherical	80	[150]
1.30	*Mammea suriga*	Antibacterial	-	Square	50	[151]
1.31	*Mentha piperita*	Antibacterial	-	Hexagonal	78	[152]
1.32	*Maytenus royleanus*	Antibacterial, Leshmenia	-	Hexagonal	30	[153]
1.33	*Musa paradisiaca (Banana)*	Antibacterial	-	Diverse	300	[154]
1.34	*Nepenthes khasiana*	Antibacterial	-	Spherical	50–80	[155]
1.35	*Nigella arvensis*	Antibacterial	-	Spherical	3–37	[156]
1.36	*Punica granatum*	Antibacterial	-	Spherical	5.20	[157]
1.37	*Pistacia integerrima*	Antibacterial	-	Granular	20–200	[158]
1.38	*Plumeria alba*	Antibacterial	-	Spherical	16–28	[159]
1.39	*Platycodon grandiflorum*	Antimicrobial	-	Spherical	15	[160]
1.40	*Rivea hypocrateriformis*	Antibacterial	-	Spherical	10–50	[161]
1.41	*Solanum nigrum*	Antibacterial	-	Spherical	50	[162]
1.42	*Salicornia brachiate*	Antibacterial	-	Polydispersed	22–35	[163]
1.43	*Solanum lycopersicums*	Antibacterial	-	Diverse	14	[164]
1.44	*Trichoderma sp*	Antibacterial	-	Pseudospheric	1–24	[165]
1.45	*Trianthema decandra L*	Antibacterial	-	Spherical, hexagonal, cuboidal	38–80	[166]
1.46	*Zingiber officinale (Ginger)*	Antibacterial	-	Spherical	5–15	[167]
1.47	*Zizyphus mauritiana*	Antibacterial	-	Spherical	20–40	[168]
2	Nanoparticles with Anticancer activity					
2.1	*Areca catechu*	Anticancer, catalyst	HeLa	Spherical	13	[123]
2.2	*Artocarpus hirsutus (Wild jack)*	Anticancer	HeLa, RKO and A549	Spherical	5–40	[169]
2.3	*Achyranthes Aspera Linn Seed*	Anticancer	HeLa (Cervical)	Spherical, hexagonal, triangular	9	[170]
2.4	*Benincasa hispida*	Anticancer	HeLa (Cervical)	Spherical	23	[126]
2.5	*Brazilian red propolis*	Anticancer	Bladder (T24) and prostate (PC-3)	Rods, triangular, pentagonal, hexagonal	8–15	[127]
2.6	*Couroupita guianensis*	Anticancer	HL-60	Cubic	27	[171,172]
2.7	*Curcuma wenyujin*	Anticancer	A498(renal carcinoma)	Spherical	200	[173]
2.8	*Ceiba pentandra (L)*	Anticancer	HCT-116 (colon cancer)	Spherical	20–48	[135]
2.9	*Corchorus olitorius*	Antiproliferative effect	(Breast) MCF-7, (colon) HCT-11, and (hepatocellular) HepG-2	Triangular, hexagonal	37–50	[174]
2.10	*Diospyros ferrea*	Anticancer	HeLa	Diverse	70–90	[136]
2.11	*Dioscorea batatas*	Cytotoxicity	B16/F10 (melanoma)	Diverse	19–56	[137]
2.12	*Dracocephalum kotschyi*	Anticancer	K562 and HeLa	Spherical	11	[140]
2.13	*Bay cedar*	Anticancer	Cervical cancer (HeLa)	Spherical	20–25	[143]
2.14	*Hevea brasiliensis*	Cytoxicity and genotoxicity	CHO-K1 cells	Spherical, triangular	50	[147]
2.15	*Justica wynaadensis*	Anticancer	(Lung cancer) A549	Spherical	30–50	[148]
2.16	*Jasminum auriculatum*	Anticancer	Cervical cancer (HeLa)	Spherical	8–37	[149]
2.17	*Lobila nicotianifolia*	Anticancer	(Lung cancer) A459	Spherical	80	[150]
2.18	*Musa paradisiaca (Banana)*	Anticancer	(Lung cancer) A459	Diverse	300	[154]
2.19	*Marsdenia tenacissima*	Anticancer	(Lung cancer) A459	Spherical	50	[175]
2.20	*Marsilea quadrifolia*	Anticancer	(Lung adenocarcinoma) (A549)	Spherical	10–40	[176]
2.21	*Mangifera indica (MI) mango peel*	Cytotoxicity	African green monkey kidney normal cells (CV-1) and fetal lung fibroblast cells (WI-38)	Round, triangular, irregular	19–45	[177]
2.22	*Nerium oleander*	Anticancer	MCF-7 (breast cancer)	Spherical	2–10	[178]
2.23	*Nepeta deflersiana*	Anticancer	(Human cervical) HeLA	Cubic	33	[179]
2.24	*Nigella arvensis*	Cytotoxicity and catalytic activities	H1299 and MCF-7	Spherical	3–37	[156]
2.25	*Orchid*	Anticancer	AMG-13 (breast cancer)	Spherical	14–50	[180]
2.26	*Punica granatum*	Anticancer	HeLa	Spherical	5–20	[157]
2.27	*Korean red ginseng*	Anticancer	(cervical), HeLa, Hep2	Spherical	3–40	[181]
2.28	*Padina tetrastromatica*	Anticancer	Liver cancer (HepG2) and lung cancer (A549)	Spherical	8–10	[182]
2.29	*Scutellaria barbata*	Anticancer	Pancreatic (PANC-1)	Spherical	154	[183]
2.30	*saffron stigma (crocin)*	Anticancer	Human breast cancer cell line (MCF-7)	Spherical	4–10	[184]
2.31	*Sargassum swartzii*	Anticancer	Human cervical carcinoma (HeLa)	Spherical	35	[185]
2.32	*Seaweed*	Anticancer	MCF-7 (breast cancer)	Cubic, spherical	20–50	[186]
2.33	*Taxus baccata*	Anticancer	Breast cells (MCF-7), cervical cells (HeLa) and ovarian cells (Caov-4)	Dispersed	20	[187]
2.34	*Wedelia trilobata*	Anticancer	HCT 15 (colon cancer)	Spherical, cubic	10–50	[188]
2.35	*Piper betle*	Cytotoxicity	HeLa and HEK293	Prism, cubic, octahedron, tetrahedron, dodecahedron, triangular	15–55	[189]
3	Nanoparticles with Antifungal activity					
3.1	*Abelmoschus* *esculentus (Okra)*	Antifungal		Crystalline	62	[190]
3.2	*Artemisia vulgaris (Mugwort)*	Larvicidal activity against Aedes larvae		Spherical, triangular, hexagonal	50–100	[191]
3.3	*Brazilian red propolis*	Antifungal		Rods, triangular, pentagonal, hexagonal	8–15	[127]
3.4	*Coreopsis lanceolate*	Detections of aflatoxins				[129]
3.5	*Carthamus tinctorius L*	Antifungal		Triangular, spherical	40–200	[131]
3.6	*Caesalpinia pulcherrima*	Antifungal		Spherical	10–50	[130]
3.7	*Bay cedar*	Antifungal		Spherical	20–25	[143]
3.8	*Helianthus annuus*	Antifungal		Polydispersed	35	[146]
3.9	*Nepenthes khasiana*	Antifungal		Spherical	50–80	[155]
3.10	*Punica granatum*	Antifungal		Spherical	5–20	[157]
3.11	*Pistacia integerrima*	Antifungal		Granular	20–200	[158]
3.12	*Rivea hypocrateriformis*	Antifungal		Spherical	10–50	[161]
3.13	*Trianthema decandra L*	Antifungal		Spherical, hexagonal, cuboidal	38–80	[166]
4	Nanoparticles with Antioxidant activity/antidiabetic activity					
4.1	*Areca catechu*	Catalyst, antioxidant	HeLa	Spherical	13.7	[123]
4.2	*Clitoria ternatea (Asian* *pigeonwings)*	Antioxidant		Spherical, triangular, hexagonal	10	[128]
4.3	*Couroupita guianensis*	Antioxidant	HL-60	Cubic	27	[171,172]
4.4	*Hoveniadulcis*	Antioxidant		Spherical	20	[145]
4.5	*Justica wynaadensis*	Antidiabetic and anti-inflammatory	(Lung cancer) A549	Spherical	30–50	[148]
4.6	*Nerium oleander*	Antioxidant	MCF-7 (breast cancer)	Spherical	2–10	[178]
4.7	*Nigella arvensis*	Antioxidant, catalytic activities	H1299 and MCF-7	Spherical	3–37	[156]

## Data Availability

Not applicable.
